# Metabolic Regulation of Immune Responses to *Mycobacterium tuberculosis*: A Spotlight on L-Arginine and L-Tryptophan Metabolism

**DOI:** 10.3389/fimmu.2020.628432

**Published:** 2021-02-09

**Authors:** Rebecca R. Crowther, Joseph E. Qualls

**Affiliations:** ^1^ Department of Pediatrics, University of Cincinnati College of Medicine, Cincinnati, OH, United States; ^2^ Division of Infectious Diseases, Cincinnati Children’s Hospital Medical Center, Cincinnati, OH, United States; ^3^ Immunology Graduate Program, University of Cincinnati College of Medicine, Cincinnati, OH, United States; ^4^ Medical Scientist Training Program, University of Cincinnati College of Medicine, Cincinnati, OH, United States

**Keywords:** tuberculosis, mycobacteria, L-arginine, L-tryptophan, amino acid

## Abstract

*Mycobacterium tuberculosis* (*Mtb*), the causative agent of tuberculosis (TB), is a leading cause of death worldwide. Despite decades of research, there is still much to be uncovered regarding the immune response to *Mtb* infection. Here, we summarize the current knowledge on anti-*Mtb* immunity, with a spotlight on immune cell amino acid metabolism. Specifically, we discuss L-arginine and L-tryptophan, focusing on their requirements, regulatory roles, and potential use as adjunctive therapy in TB patients. By continuing to uncover the immune cell contribution during *Mtb* infection and how amino acid utilization regulates their functions, it is anticipated that novel host-directed therapies may be developed and/or refined, helping to eradicate TB.

## Introduction

Tuberculosis (TB), caused by *Mycobacterium tuberculosis* (*Mtb*), is the leading worldwide cause of death due to a single infectious agent (1). Approximately one quarter of the world’s population is infected with *Mtb*, with 10 million new infections each year (1). In 2019, *Mtb* claimed nearly 1.3 million deaths, with over 250,000 deaths in HIV-positive patients – representing a greater than 20% reduction in TB-related deaths since 2000 (1). Progress in ending the TB epidemic is slow, however, and at this rate TB is unlikely to be eradicated this century (2). The COVID-19 pandemic has significantly curtailed this progress and is estimated to result in an additional 400,000 TB-related deaths in 2020 (1). Additionally, drug-resistant *Mtb* strains continue to pose a public health problem, especially in developing countries with the highest rates of infection (1, 2). While the live attenuated *M. bovis* Bacille Calmette-Guérin (*Mb* BCG) vaccine is administered to newborns in TB endemic countries, little protection is formed against pulmonary TB (3). Additionally, *Mb* BCG cannot be administered to immunocompromised patients due to the high risk of disseminated infection (1–3). As such, new strategies are urgently needed to end the fight against TB.

In response to infection, immune cells undergo metabolic changes. Following LPS stimulation, macrophages utilize aerobic glycolysis to generate the energy needed to fuel their effector functions (4). By contrast, while *Mtb*-infected inflammatory macrophages seeming rely on glycolysis, *Mtb*-infected alveolar macrophages utilize fatty acid β-oxidation for energy (5). Additionally, following activation T cells undergo metabolic reprogramming, switching from a naïve state where energy is derived mainly from the TCA cycle and oxidative phosphorylation to an activated state dominated by aerobic glycolysis (6). Thus, precise alterations in metabolic function depend on the pathogen and cell type involved. Amino acid metabolism is often affected following infection. Amino acids are the building blocks of proteins, but also regulate cellular responses *via* nutrient sensor signaling or serve as anapleurotic precursors for energy-producing pathways, such as the tricarboxylic acid (TCA) cycle. Therefore, adjustments in amino acid concentrations regulate how immune cells respond to infection.

Targeted metabolomics studies have proposed metabolite changes, particularly in amino acid abundance, may serve as biomarkers following *Mtb* infection. Multiple studies have observed decreased L-citrulline (L-CIT) and L-ornithine (L-ORN) – metabolites of L-arginine (L-ARG) – in the sera of active TB patients compared to healthy controls (7–9). Interestingly, following antibiotic treatment, L-CIT and L-ORN levels in TB patients increase to those of healthy controls (8). Additionally, active TB patients display decreased L-tryptophan (L-TRP) and increased levels of its metabolites, including L-kynurenine (KYN), in their sera and urine as compared to healthy controls (7–11). In fact, metabolite tracking may predict a patient’s TB status. One study found just 20 serum metabolites, 11 of which were amino acids or derivatives, were required to discriminate active TB patients from healthy controls (7). When tracking household contacts of TB patients, amino acid alterations in the serum could discriminate between patients who later developed TB and those who remained healthy (12).

Given these data, it is important to understand how amino acids contribute to immune cell function following *Mtb* infection. Here, we review immune responses during *Mtb* infection and immune cell metabolism of two key amino acids: L-ARG and L-TRP.

## Immune Response to *Mycobacterium tuberculosis*

### Macrophages

**Figure 1:**
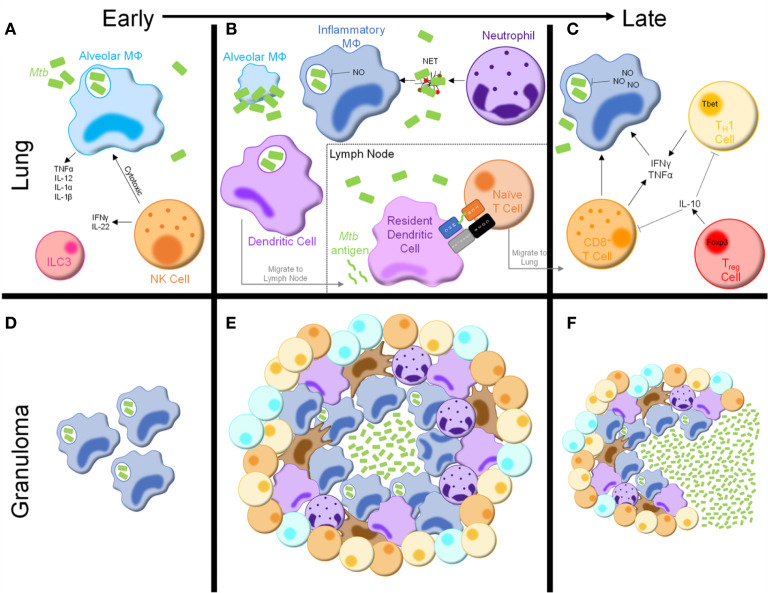
Immune response to *Mycobacterium tuberculosis*. **(A)** Following inhalation, *Mtb* bacilli are phagocytosed by alveolar macrophages in the lung, resulting in cytokine and chemokine production. NK cells are innate lymphocytes that are cytotoxic to infected alveolar macrophages and produce inflammatory cytokines. Group 3 innate lymphoid cells (ILC3s) are also present in the lung early following *Mtb* infection. **(B)** As infection progresses, alveolar macrophages are overrun with *Mtb* bacilli and undergo cell death. Inflammatory macrophages, neutrophils, and dendritic cells are recruited from the bloodstream. Inflammatory macrophages produce mycobactericidal nitric oxide (NO) and phagocytose *Mtb*-containing neutrophil extracellular traps (NETs), secreted by neutrophils. Dendritic cells phagocytose *Mtb* bacilli and migrate to the draining lymph node, where they secrete *Mtb* antigens to be presented by resident dendritic cells, initiating naïve T cell activation. **(C)** After migrating to the lymph node, activated T cells secrete inflammatory cytokines, including interferon (IFN)γ and tumor necrosis factor (TNF)α, which further stimulate macrophage anti-mycobacterial activity. Activated CD8^+^ T cells are also cytotoxic to infected macrophages. T_reg_ cells produce cytokines, such as IL-10, to inhibit the activity of other lymphocytes in the lung. **(D–F)** Concurrently, granulomas begin to develop in the lung. **(D)** Early granulomas consist of aggregations of infected phagocytes. **(E)** Mature granulomas consist of a core of *Mtb* bacilli, infected macrophages, and multinucleated giant cells surrounded by more macrophages, neutrophils, dendritic cells, and fibroblasts. The outside of the granuloma consists of T and B cells, forming a lymphocytic cuff. **(F)** As infection progresses and potentially following some degree of immunosuppression the granulomas begin to break down, leading to dissemination of *Mtb* bacilli and reactivation of TB disease.

Upon inhalation, *Mtb* bacilli are phagocytosed by alveolar macrophages (**Figure 1A**), resident phagocytes within alveoli (13, 14). Alveolar macrophages make up the majority of mycobacteria-laden cells in the lung during early *Mtb* infection, with neutrophils not appearing until 10-14 days post-infection (14–16). Additionally, accumulation of group 3 innate lymphoid cells (ILC3s) parallels that of alveolar macrophages and precedes infiltration of inflammatory macrophages (17).

Alveolar macrophages express numerous pattern recognition receptors involved in *Mtb* recognition, stimulation of which induces an inflammatory response and recruitment of additional innate immune cells, including neutrophils, inflammatory macrophages, and dendritic cells (DCs) (16, 18, 19). Although alveolar macrophages rapidly phagocytose *Mtb* bacilli, the immune response is slow, contributing to impaired mycobacterial clearance. *Mtb* bacilli inhibit a variety of effector responses, including phagolysosome fusion and cytokine production, resulting in mycobacterial overload, cell death, and widespread distribution of infection throughout the lung (**Figure 1B**) (20–23).

The myeloid population responding to *Mtb* infection is diverse, marked by production of various anti-mycobacterial effectors including nitric oxide (NO), tumor necrosis factor (TNF) α, interleukin (IL)-12, IL-1α, and IL-1β, all of which are critical for infection control (24–26). An abundance of literature supports a dual role for macrophages as the main effector killing cells and a growth niche for mycobacteria. Inflammatory macrophages, as the main reservoir for *Mtb* growth, are the most well studied. Like their alveolar counterparts, inflammatory macrophages are impaired in combating *Mtb* infection due to inhibition of effector functions, as well as *Mtb* adaptations to a harsh intracellular environment (20–22).

Despite this inhibition, inflammatory macrophages harness multiple pathways to restrict *Mtb* growth. Combined TLR2 and IFNγR stimulation leads to transcription of *Nos2*, which encodes inducible NO synthase (iNOS), resulting in NO production (**Figure 1B**) (19). In mice, NO positively correlates with *Mtb* killing and mice lacking NO synthesis fail to control mycobacterial infections, succumbing even to *Mb* BCG (27–30). iNOS and NO production have been detected in human macrophages *in vitro* and the lungs of *Mtb*-infected patients (31–34). The contribution of NO in the pathogenesis of human *Mtb* infection, however, is highly debated. Some studies have found NO production does not play a central role in control of human *Mtb* infection, while others suggest clearance of *Mtb* is NO-dependent (32, 35–39). Nevertheless, the presence of iNOS and NO in the *Mtb*-infected lung suggests NO is involved in human immune responses to *Mtb* infection, albeit not entirely similar to those in mice.

While NO is a predominant mycobactericidal mechanism, macrophages also utilize nutrient limitation, reactive oxygen species, and apoptotic and autophagic pathways for *Mtb* elimination (19, 40). However, *Mtb* harnesses various inhibitory mechanisms to prevent its clearance *via* these mechanisms (23, 41). Therefore, it is evident macrophages play an important role in combating *Mtb* infection, but their efforts are thwarted by microbial adaptations.

### Neutrophils

Neutrophils are short-lived innate immune cells that rapidly migrate to the lungs upon *Mtb* infection (**Figure 1B**) (42). Neutrophils phagocytose *Mtb* bacilli, yet evidence of their ability to kill mycobacteria is conflicting (43–45). While studies have shown neutrophil-derived molecules are mycobactericidal, neutrophils display poor antimycobacterial activity and may contribute to destruction of the lung parenchyma (43, 44, 46–49). Upon activation, neutrophils release neutrophil extracellular traps (NETs) composed of nuclear proteins and proteases. Nucleosomes and elastase – both found within NETs – have been detected in plasma of active TB patients, and *Mtb* can induce NET formation *in vitro* (50–53). Despite evidence that *Mtb*-induced NETs are phagocytosed and stimulate macrophage cytokine production, NETs also provide a niche for *Mtb* growth (52, 53). Therefore, a clear role for neutrophil mediated *Mtb* defense has not yet been elucidated.

Neutrophil activity enhances anti-mycobacterial responses in peripheral immune cells. Infected neutrophils interact with and increase DC antigen presentation and T cell stimulatory capacity (54). Following administration of a *M. smegmatis*-derived vaccine in mice, neutrophil depletion decreased T cell responses and increased mycobacterial burden upon *Mtb* challenge (55). As such, neutrophils may play an important role in coordinating innate and adaptive immune responses following *Mtb* infection.

### Natural Killer Cells

Natural killer (NK) cells – cytolytic innate lymphocytes recognize ligands present in the *Mtb* cell wall and molecules expressed by *Mtb*-infected phagocytes, leading to cytolytic killing of infected cells (**Figure 1A**) (56–58). Additionally, NK cells secrete IFNγ and IL-22 following *Mtb* infection, stimulating phagosome maturation in *Mtb*-infected macrophages (59). Recent studies have identified changes in both human and mouse NK cells following *Mtb* infection, resulting in decreased NK cell number and functionality (60–62). Taken together, NK cells are likely an important immune contributor during *Mtb* infection, but their definitive role remains to be elucidated.

### Dendritic Cells

DCs are essential in linking innate and adaptive immunity. In mice, DCs make up one of the largest proportions of *Mtb*-infected phagocytes by 4 weeks post-infection (63). Patients with DC defects are highly susceptible to disseminated mycobacterial infections, likely due to impaired adaptive immune activation (64). To initiate adaptive immunity, DCs phagocytose *Mtb* bacilli and migrate to draining lymph nodes to present antigenic peptides (**Figure 1B**) (13, 63, 65). Interestingly, this priming requires live *Mtb* bacilli within the lymph node (63, 65). However, *Mtb*-laden DCs are poor antigen presenters and instead secrete soluble, unprocessed *Mtb* antigens, which are presented by uninfected lymph node resident DCs (63, 66, 67). While this process circumvents inefficient antigen presentation by *Mtb*-infected DCs, it may also lead to decreased T cell priming (67, 68). Regardless, DCs serve a central role in priming the adaptive immune response following *Mtb* infection.

### CD4^+^ T Cells

CD4^+^ T cells are essential for anti-mycobacterial immunity, as HIV/AIDS patients with low CD4^+^ T cell counts and animal models lacking CD4^+^ T cells quickly succumb to infection, even with attenuated mycobacterial strains (69–71). Naïve T cell activation is delayed following infection due to a variety of evasive mechanisms harnessed by *Mtb* (67, 72). In mouse models, antigen-specific CD4^+^ T cells are detected in the draining mediastinal lymph nodes and lungs 2 and 3 weeks post-*Mtb* infection, respectively (19). In non-human primates, antigen-specific CD4^+^ T cells are not detected in the lungs until 4 weeks post-infection (73). Adoptive transfer studies indicated CD4^+^ T cell activation in the lymph node does not begin until 7 days post-infection, despite an abundance of antigen-specific cells (74). These findings indicate a combination of ineffective antigen presentation and immune evasion significantly delay priming of anti-*Mtb* adaptive immunity.

Activated CD4^+^ T cells migrate to the infected lung and interact with innate cells to reinforce anti-*Mtb* immune responses (**Figure 1C**). IFNγ- and TNFα-producing CD4^+^ T_H_1 cells are required for optimal anti-mycobacterial responses, as humans and mice with defects in the IFNγ/IL-12/T_H_1 axis are highly susceptible to mycobacterial infection (75–82). Mice lacking T_H_17 immunity display increased *Mtb* burden following infection (83, 84). Adoptive transfer of antigen-specific T_H_17 cells into T cell-deficient mice prolongs survival, even when IFNγ-deficient, suggesting T_H_17 cells act independently of T_H_1 cells to control *Mtb* infection (85). Despite this, T_H_17 responses are negligible in active TB patients, indicating work is still needed to determine the impact of T_H_17 immunity in human *Mtb* infection (86). Thus, while the T_H_1 cells are essential, further investigation into other CD4^+^ T cell subtypes is required to better understand the anti-mycobacterial immune response.

IFNγ is a main driver of *Mtb* growth restriction and prevention of extrapulmonary dissemination through induction of iNOS, inflammatory cytokines, and antimicrobial peptides in infected macrophages (87). Despite its protective role, IFNγ has been implicated in the pathogenesis of prolonged *Mtb* infection as it promotes cavitation and destruction of lung tissue (88, 89). As such, T_H_1 activity is restricted by IL-10, an inhibitory cytokine produced chiefly by macrophages and Foxp3^+^ T regulatory (T_reg_) cells (**Figure 1C**) (90–92). T_reg_ expansion has been detected in *Mtb*-infected macaques and humans (93–96). T_H_1 cells co-secreting IFNγ and IL-10 have also been detected in bronchoalveolar lavage fluid from active TB patients and mice with high infectious doses, though the contribution of these cells remains unclear (97, 98). In mice, T_reg_ depletion enhances mycobacterial clearance at the expense of increased pulmonary immunopathology, while T_reg_ expansion dampens T_H_1 responses (99–101). Taken together, T_H_1 immunity, while critical for *Mtb* clearance, is dampened by T_reg_ cells in order to preserve lung architecture and function following *Mtb* infection.

### CD8^+^ T Cells

Loss of CD8^+^ T cell responses impairs anti-mycobacterial immunity in animal models (102–104). Though an exact role remains unclear, CD8^+^ T cells prevent TB reactivation *via* cytokine production, perforin-mediated lysis of *Mtb*-infected phagocytes, and secretion of cytotoxic granules for direct killing of intracellular *Mtb* (**Figure 1C**) (105–107). TNFα inhibitors selectively deplete a subset of granulysin-secreting memory CD8^+^ T cells, which may explain why patients with autoimmune diseases prescribed anti-TNFα drugs are more susceptible to mycobacterial infections (108). Studying the role of CD8^+^ T cells in *Mtb* infection has been difficult, however, as CD8^+^ T cells cannot recognize *Mtb*-infected macrophages to the same extent as their CD4^+^ counterparts (109). While more work is needed, these studies show CD8^+^ T cells help control mycobacterial infection.

### B Cells

Despite being an intracellular pathogen, growing evidence indicates humoral immunity is essential for anti-*Mtb* immune responses. Anti-*Mtb* antibodies have been detected in humans exposed to *Mtb* and provide protection in mouse models (110). However, the antibody pool in sera of active TB patients is small and limited to membrane-associated and extracellular antigens (111). Interestingly, Fc glycosylation of anti-*Mtb* antibodies differs depending on the stage of infection and can discriminate between latent and active TB patients (112, 113). Antibodies secreted in latent TB promote increased NK cell responses and inflammasome activation of human monocyte derived macrophages *in vitro* (112). Additionally, human monocyte derived macrophages cultured with antibodies from latent TB patients displayed decreased mycobacterial burden as compared to those cultured with antibodies from active TB patients (112). These findings are complemented by reports that IgG glycosylation of anti-*Mtb* antibodies is required to observe protection upon transfer into mouse models (114, 115). Still, the role of humoral immunity against *Mtb* requires additional investigation.

### The Granuloma

Despite a coordinated immune response, patients are generally unable to clear *Mtb* infection. As such, the immune system walls off *Mtb* bacilli in granulomas, a hallmark of human TB disease (**Figures 1D–F**). Granulomas are characterized by a central core of *Mtb*-infected multinucleated giant cells and uninfected macrophages at varying stages of differentiation, surrounded by macrophages, neutrophils, DCs, and fibroblasts, along with a lymphocytic cuff composed of T and B cells (116–119). Granulomas can contain *Mtb* infection for decades. However, hypoxia causes the core necrosis, allowing for dissemination and reactivation of pulmonary TB infection and/or seeding in extrapulmonary sites (120, 121). Additionally, the granuloma structure precludes drug penetration and elimination of *Mtb* bacilli (122). Thus, while protective against active disease, granulomas present a therapeutic issue contributing to the difficulty of eradicating TB.

The immune response against *Mtb* is multifaceted and highly dependent on the cell’s metabolic state and ability to acquire nutrients. Minor alterations in immune cell metabolism have the potential to fundamentally change outcomes throughout *Mtb* infection. Changes in carbohydrate and fatty acid metabolism following *Mtb* infection, particularly in macrophages, have been well described and reviewed (5, 118, 123, 124). Amino acids can impact cellular nutrient signaling and be utilized by immune cells in a variety of metabolic pathways. For example, L-glutamine can signal nutrient sufficiency through mTOR or be oxidized and converted to other energy sources like citrate, which can enter the TCA cycle or be utilized for fatty acid synthesis (123). Meanwhile, L-serine, which plays a main role in 1 carbon metabolism *via* the folate cycle, has been shown to support macrophage functions through mTOR activation (125). Here we will focus on L-ARG and L-TRP metabolism in immune cells following *Mtb* infection, and how their acquisition and utilization change over the disease course.

## L-Arginine Metabolism

L-ARG is an amino acid obtained from the diet, generated *via de novo* synthesis, or scavenged following protein turnover (126). At homeostasis, the intestinal-renal axis of L-ARG synthesis maintains the body’s L-ARG supply (127, 128). However, in early life, during chronic infection or inflammation, or following renal or intestinal injury, synthesis is not sufficient to meet the body’s needs, classifying L-ARG as a semi-essential amino acid (126). L-ARG is also part of the urea cycle, a pathway utilized to eliminate nitrogenous waste products. Patients with most urea cycle disorders exhibit impaired *de novo* synthesis, so L-ARG is considered essential (126).

**Figure 2:**
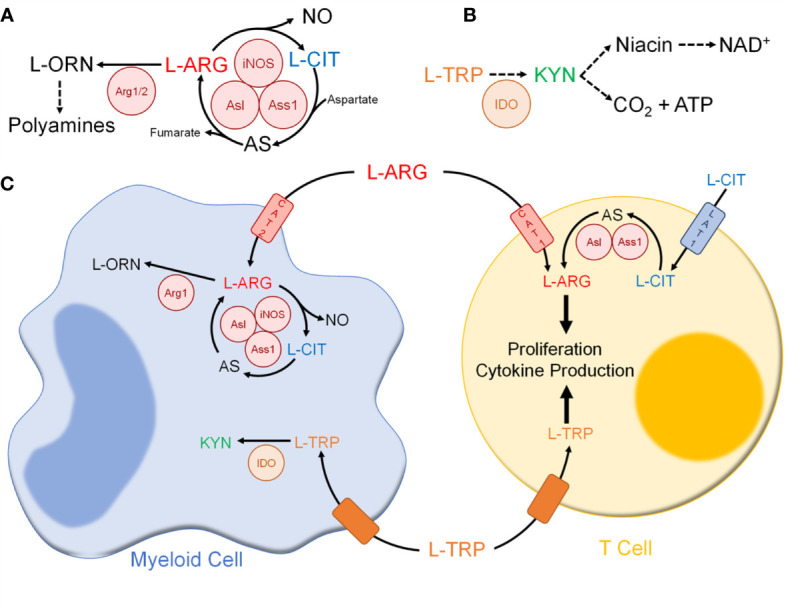
Immune cell amino acid metabolism following mycobacterial infection. **(A)** L-ARG metabolism pathway in immune cells. **(B)** L-TRP metabolism pathway in immune cells. **(C)** Amino acid metabolism between myeloid and T cells. Abbreviations: L-ARG, L-arginine; L-CIT, L-citrulline; L-ORN, L-ornithine; iNOS, inducible nitric oxide synthase; Ass1, argininosuccinate synthase 1; Asl, argininosuccinate lyase; Arg1/2, arginase 1/2; L-TRP, L-tryptophan; KYN, L-kynurenine; IDO, indolamine 2,3-dioxygenase; CAT1, cationic amino acid transport 1; CAT 2, cationic amino acid transporter 2; LAT1. L-type amino acid transporter 1.

L-ARG is the sole substrate for NO synthase enzymes (126). In myeloid cells, L-ARG is converted to NO *via* iNOS, forming L-citrulline (L-CIT) as a by-product (**Figure 2A**) (129, 130). L-CIT is a noncanonical amino acid utilized in the urea cycle and found in peptides/proteins following posttranslational modification of peptidyl-L-ARG (127, 130). In some cells, including immune cells, L-CIT serves to generate L-ARG. Through the sequential activity of argininosuccinate synthase 1 (Ass1) and argininosuccinate lyase (Asl), L-CIT is metabolized to form L-ARG (130). These reactions require L-aspartate and generate L-fumarate, which are intermediates in the TCA cycle. L-ARG can also be metabolized by arginases to form L-ornithine (L-ORN), a noncanonical amino acid formed in the urea cycle and a polyamine precursor (126, 131). There are two known arginase isoforms – cytosolic arginase 1 (Arg1) and mitochondrial arginase 2 (126).

L-ARG is also a precursor of creatine, L-proline, L-glutamate, and agmatine (126). Additionally, L-ARG regulates the protein kinases and cellular nutrient sensors general control nonderepressible 2 (GCN2) and mammalian target of rapamycin complex 1 (mTORC1) (132). The roles of GCN2 and mTORC1 in immune cells, and their regulation by L-ARG, have been reviewed elsewhere (133–136) and will not be discussed here.

### L-arginine Metabolism and Implications on the Anti-Mycobacterial Immune Response

#### Macrophages

Macrophages are often characterized as M1 (“classically activated”) or M2 (“alternatively activated”) based on their metabolic state and effector molecules. In fact, L-ARG utilization has often been used to classify macrophages, where iNOS or Arg1-expressing macrophages are considered “M1” or “M2”, respectively (118, 124). Following mycobacterial infection, however, macrophages co-express iNOS and Arg1 (137–139). Thus, macrophages that respond to *Mtb* infection do not concisely fit within the “M1” versus “M2” binary with respect to L-ARG utilization.

In mice, macrophages have been shown to utilize L-ARG to fuel NO production *via* iNOS (**Figure 2**) (124, 129). However, following *Mtb* infection extracellular L-ARG is not sufficient to sustain NO synthesis. As such, macrophages convert L-CIT into L-ARG, which is instrumental for continued NO production (139, 140). Amino acid tracing showed regeneration of L-ARG from L-CIT preferentially feeds into NO synthesis *via* iNOS, as opposed to L-ORN synthesis *via* Arg1 (140). This corresponds with previous studies indicating structural colocalization of NOS enzymes with Asl, one of the L-ARG synthesis enzymes, during systemic NO production (141). Evidence of colocalization of Ass1, Asl, and iNOS has not been definitively shown, though a combination of immunoprecipitation and tandem mass spectrometry suggests such a complex is formed within RAW 264.7 murine macrophages (141). Thus, macrophage L-ARG synthesis from L-CIT likely serves to fuel NO synthesis. Still, there are likely additional macrophage anti-mycobacterial mechanisms that require a sufficient L-ARG supply, and investigation outside of NO production will be beneficial since NO does not play a dominant role in human macrophages.

Immune cell L-ARG synthesis is vital for controlling mycobacterial burden. Bone marrow chimeric mice with hematopoietic cells from Ass1 hypomorphs were unable to control *Mtb* infection as compared to controls (139). Additionally, mice with a conditional hematopoietic or myeloid deletion of Asl or Ass1 displayed increased burden following *Mb* BCG or *Mtb* infection (142). Therefore, L-ARG and its synthesis contribute to macrophage defense following mycobacterial infection.

Arg1-expressing macrophages are present following *Mtb* infection in humans, non-human primates, and mice (138, 143–145). Though L-ORN is a polyamine precursor, these metabolites have little effect on *Mtb* growth *in vitro* (145). Instead, by synthesizing L-ORN, Arg1 depletes the microenvironment of L-ARG (**Figure 2**) (129). Arg1 expression occurs alongside upregulation of CAT-2B, the high-affinity isoform of L-ARG transporter CAT-2 (146, 147). Though iNOS has been shown to have a higher affinity for L-ARG, Arg1 has greater maximal activity, and as such imported L-ARG is preferentially shuttled for L-ORN synthesis rather than NO production (148). In mice co-infected with *Schistosoma mansoni* and *Mtb*, Arg1 impaired mycobacterial control and worsened lung pathology (149). A mouse model lacking Arg1 in hematopoietic cells displayed increased macrophage NO production and enhanced mycobacterial clearance (137). Thus, Arg1-expressing macrophages likely play an inhibitory role, limiting NO production and *Mtb* clearance.

Arg1-expressing macrophages are found in granulomas from *Mtb*-infected patients and non-human primates (138, 143). Using an intradermal *Mtb* infection model in *Nos2^-/-^* mice, Arg1-expressing macrophages were detected in granulomas (145). Interestingly, loss of Arg1 in hematopoietic cells of *Nos2^-/-^* mice resulted in more enlarged, necrotic granulomas and increased mycobacterial burden (145). Additionally, presence of Arg1 in granulomas correlated with decreased T cell proliferation (145). Thus, it is likely Arg1 regulates T cell-mediated lung immunopathology following *Mtb* infection.

#### Neutrophils

Human neutrophils store and release Arg1 upon activation *in vitro* (150). In sepsis patients, circulating neutrophil counts positively correlate with plasma Arg1 activity (151). Following phagocytosis of fungi, Arg1 is upregulated in neutrophils (152). Airway supernatant from cystic fibrosis patients suppresses T cell responses *in vitro*, which was reversed following addition of Arg1 inhibitor N-ω-Hydroxy-L-norarginine (153). This was attributed to neutrophil Arg1 release, as neutrophil counts positively correlated with airway supernatant Arg1 activity (153). Thus, multiple mechanisms of neutrophil Arg1-mediated immune regulation exist, yet neutrophil Arg1 activity in *Mtb* infection still requires investigation.

The role of neutrophil L-ARG synthesis has not been studied in *Mtb* infection. Our work investigating myeloid L-ARG synthesis following mycobacterial infection utilized Lyz2-cre for conditional deletion, also targeting neutrophils (142). However, the role of neutrophil-specific L-ARG metabolism following mycobacterial infection has not been addressed and warrants further investigation.

#### NK Cells

NK cells require L-ARG for proliferation, expression of activating receptors, and IFNγ production *in vitro* (154). Human NK cells constitutively express endothelial NOS, inhibition of which impairs cytotoxicity (155, 156). Like T cells, NK cells are inhibited by myeloid-derived Arg1 (157–159). In hepatitis C virus infection, Arg1-producing myeloid-derived suppressor cells decreased NK cell IFNγ production through mTOR inhibition, which was reversed upon *in vitro* L-ARG supplementation (158). Despite this, neither NK cell L-ARG metabolism nor Arg1-mediated inhibition have been explored following *Mtb* infection.

#### Dendritic Cells

Mouse bone marrow derived DCs (BMDCs) express Arg1 during development and have active GM-CSF-dependent L-ARG metabolism (160, 161). Additionally, mouse BMDCs grown without L-ARG decreased MHC II surface expression, while MHC I expression was unaffected (162). Thus, L-ARG is involved in BMDC development, though more thorough investigation is needed.

Little is known about DC L-ARG metabolism following infection. TNF/iNOS-producing DCs are one of the main inflammatory myeloid cell types present following bacterial and parasitic infections, though they have not been investigated following *Mtb* infection (163–165). Additionally, *Giardia duodenalis*-infected human monocyte-derived DCs decrease expression of the co-stimulatory molecule CD86 in the absence of L-ARG (166). However, DC L-ARG metabolism during *Mtb* infection has not yet been investigated.

#### T Cells

T cells rely on L-ARG for cell cycle progression, proliferation, and effector functions (**Figure 2**) (167–172). T cells express the L-ARG synthesis enzymes, with Asl and Ass1 induced upon *in vitro* activation (173, 174). Functions requiring L-ARG can be rescued by L-CIT when L-ARG is limiting *in vitro* (173–175). Human CD4^+^ T cells utilize CAT-1 and LAT-1 to import L-ARG and L-CIT, respectively (174, 176).

T cell responses are suppressed when L-ARG is limiting. Myeloid Arg1 has been implicated in T cell suppression observed following mycobacterial infection, vaccination, and cancer (**Figure 2**) (153, 177–180). Following *in vitro* stimulation with heat-killed *Mb* BCG, macrophage Arg1 suppressed T cell proliferation, which was restored in cultures supplemented with L-CIT (173). These data suggest L-ARG synthesis from L-CIT may circumvent T cell suppression following L-ARG depletion. Still, mice with a T cell-specific loss of L-ARG synthesis did not display impaired mycobacterial clearance with either *Mtb* Erdman or *Mb* BCG, yet displayed a slight, although significant, decrease in CD4^+^ T cell accumulation in the lung and draining lymph nodes, compared to controls (173). Given the accumulation impairment, mycobacterial burden and/or T cell mediated lung immunopathology should be investigated during multiple stages of infection.

#### B Cells

L-ARG deficiency impairs B cell maturation at the pro- to pre-B cell stage (181). Arg1-treated 697 pre-B lymphoblastic cells displayed increased cell death, which was reversed or attenuated upon addition of L-ARG or L-CIT, respectively (182). Therefore, it is likely B cells express L-ARG synthesis machinery, although this has not been definitively determined. Regardless, the impact of B cell L-ARG utilization and metabolism have not yet been explored following *Mtb* infection.

#### L-Arginine Supplementation in Tuberculosis Treatment

Given L-ARG is required for optimal immune cell function, L-ARG supplementation has been attempted *in vitro* and *in vivo* and has been considered as a potential vaccine adjuvant or therapeutic (139, 140, 183–187). L-ARG supplementation has been investigated in the treatment of active TB with mixed results. Some studies have shown oral L-ARG decreases constitutional symptoms and increases sputum conversion in active TB patients (188, 189). However, another study in which active TB patients were given a high L-ARG diet found no benefit when compared to normal diet, even though HIV-positive patients showed an increased cure rate (190). Regardless, oral L-ARG is subject to metabolism in the small intestine, resulting in excess water and electrolyte secretion and severe gastrointestinal distress, making it unlikely for oral L-ARG supplementation to be used clinically in TB treatment (191).

L-CIT presents a potential therapeutic alternative as it increases circulating L-ARG and NO following oral supplementation and has not been shown to induce gastrointestinal side effects (191–194). Additionally, use of L-CIT as an adjuvant improved antibody production following viral vaccination in mice (195). Addition of L-ARG alone or with L-CIT, however, did not increase antibody titers (195). This indicates L-CIT supplementation may be considered for boosting immune function when L-ARG is not recommended.

In summary, L-ARG plays an important role in immune cells following mycobacterial infection. In macrophages, L-ARG is needed to fuel NO synthesis and its synthesis from precursor L-CIT is required to control mycobacterial infection. Additionally, Arg1-expressing macrophages likely control T cell mediated immunopathology following *Mtb* infection *via* L-ARG depletion. T cells require L-ARG to fuel their effector functions, setting up an effective competition for nutrients with myeloid cells (**Figure 2C**). The requirement for L-ARG in both macrophages and T cells has been extensively studied, and likely serves as an immunoregulatory mechanism following *Mtb* infection. The role of L-ARG in other myeloid cells, including neutrophils and dendritic cells, as well as NK cells and B cells has not been extensively studied in the context of *Mtb* infection. Additionally, the use of L-ARG and/or L-CIT as potential therapeutic or preventative approaches should be further investigated in the context of *Mtb* infection and vaccination.

## L-Tryptophan Metabolism

L-TRP is an essential amino acid obtained from the diet (196). However, in cases where L-TRP cannot be properly absorbed or is in low abundance, L-TRP may be in short supply (196). Proteolysis allows for recycling of L-TRP to meet cellular nutrient requirements, but the contribution of protein catabolism to L-TRP plasma concentrations is unclear (197).

When in excess, L-TRP is metabolized to form niacin (**Figure 2B**). In fact, L-TRP is second only to niacin itself as the body’s main source of NAD^+^, a reducing equivalent that is required for many metabolic reactions, such as glycolysis and the TCA cycle (198). To form NAD^+^, L-TRP is metabolized *via* the KYN pathway (196, 199). In hepatocytes, steroids and excess L-TRP upregulate tryptophan 2,3-dioxygenase (TDO), which converts L-TRP to N-formylkynurenine, a KYN precursor (196, 200). In innate immune cells, TLR and IFNγR ligation increase indoleamine 2,3-dioxygenase (IDO), which catalyzes the same reaction (196, 200). Unlike TDO, IDO allows for more promiscuous substrate binding, including 5-hydroxytryptophan, serotonin, and D-tryptophan (201). While both require oxygen, TDO requires heme-associated molecular oxygen and IDO requires superoxide anions (199, 201). Of the enzymes that metabolize L-TRP through the KYN pathway, only IDO is present and active in immune cells.

Two IDO isoforms, IDO1 and IDO2, are expressed by antigen presenting cells, epithelial cells, vascular endothelial cells, and neoplastic cells (199, 200). Discriminating between contributions of IDO1 and IDO2 is difficult, as available inhibitors are not isoform-specific (196, 199, 202). While knockout mouse lines for each isoform exist, only the *Ido1^-/-^* line is commercially available (203, 204). As such, we will refer to IDO1 and IDO2 collectively as “IDO” and provide information about specific isoforms when available.

L-TRP also forms serotonin, melatonin, and picolinate (**Figure 2B**) (196, 200). Like L-ARG, L-TRP regulates mTOR and GCN2; this has been reviewed elsewhere and will not be discussed here (133–136).

### L-tryptophan Metabolism and Implications on the Immune Response to *Mycobacterium tuberculosis*

IDO activity, measured by L-TRP/KYN ratio, can determine TB severity. Active TB patients with low L-TRP/KYN ratios (high IDO activity), have poorer outcomes compared to those with low IDO activity (205). Additionally, active TB patients with pleurisy had increased pleural fluid IDO activity compared to patients with non-infectious pleuritis (206). Active TB patients also exhibit increased sputum IDO compared to patients with other lung diseases (207). In HIV^+^ patients, plasma IDO activity is increased upon *Mtb* co-infection and could be detected 6 months prior to TB diagnosis (208). Thus, measuring IDO activity may have prognostic value in TB diagnosis.

#### Macrophages

Upon *Mtb* infection, macrophages expression of IDO is IFNγ-dependent (**Figure 2**) (209, 210). In macaques, lung IDO expression is limited to macrophage-rich areas of granulomas (211). Some have postulated macrophage IDO-mediated L-TRP depletion exerts an anti-microbial effect; however *Mtb* can synthesize L-TRP, making this an unlikely anti-mycobacterial mechanism (212–214). Macaques treated with IDO inhibitor 1-methyl-DL-tryptophan (1-MT) showed decreased mycobacterial burden and lung pathology compared to controls following *Mtb* CDC1551 infection, while there was no difference in mycobacterial burden between *Ido1* ^-/-^ and wild-type mice (209, 215). Thus, a central anti-mycobacterial role for IDO has yet to be elucidated.

IDO forms metabolites that bind and activate the aryl hydrocarbon receptor (AHR), a ligand-regulated transcription factor that modulates immune activity. Following *Mtb* H_37_R_v_ infection, *Ahr* ^-/-^ mice displayed increased systemic mycobacterial burden, attributed to loss of myeloid cell AHR (216). Macrophage AHR activation induces IL-1β and IL-23 transcription, supporting IL-22 producing lymphocytes (217). Thus, the role of IDO is not limited to L-TRP depletion, as L-TRP metabolites modulate immune responses through AHR activation.

#### Neutrophils

While unexplored in TB, L-TRP metabolism in neutrophils has been examined in other infection models. Neutrophil IDO expression is IFNγ-dependent (218). Following *Pseudomonas aeruginosa* infection, KYN blunted neutrophil-mediated bacterial killing by acting as a reactive oxygen species scavenger (219). In mice, KYN impaired neutrophil chemotaxis in an AHR-dependent manner following uropathogenic *Escherichia coli* infection (220). Thus, IDO activity inhibits neutrophil responses, and its contributions to neutrophil function following *Mtb* infection require further investigation.

#### NK Cells

L-TRP metabolism in NK cells is unexplored during *Mtb* infection. In NK92 MI cells, KYN induces apoptosis, an effect dependent upon the presence of reactive oxygen species (221). In a mouse B16 melanoma model, 1-MT administration decreased NK cell cytotoxicity, reducing their ability to restrict tumor size (222). Following *Paracoccidioides brasiliensis* infection, *Ido1* ^-/-^ mice displayed decreased lung NK cell expansion as compared to controls (223). Thus, the available data are limited, requiring further exploration to elucidate the role of L-TRP metabolism in general NK cell biology and following *Mtb* infection.

#### Dendritic Cells

DCs express IDO in an IFNγ-dependent manner (218). BMDCs lacking IDO supported increased proliferation of mycobacterial-specific CD4^+^ T cells *in vitro* (209). Thus, DC IDO expression likely plays a tolerogenic role following *Mtb* infection. Interestingly, a feedback loop involving Arg1 and IDO1 has been uncovered in DCs (224). In mouse CD11c^+^ splenic DCs, TGF-β induces Arg1 expression, followed by Ido1 upregulation (224). Interestingly, increased Ido1 expression was lost in Arg1-deficient DCs, suggesting Arg1 is required for Ido1 upregulation (224). This was mediated by spermidine, a polyamine, *via* activation of Src kinase (224). Thus, formation of tolerogenic DCs requires a coordinated effort between the L-ARG and L-TRP metabolic pathways. This relationship should be further explored in other myeloid cells and in *Mtb* infection.

#### T Cells

L-TRP is essential for supporting T cell functions, which are dampened by myeloid IDO activity (**Figure 2**) (225–227). When co-cultured with human CD3^+^ T cells, pleural fluid from active TB patients inhibited cell cycle progression, T_H_1 polarization, and cytokine production (228). The addition of 1-MT reversed the effect on cytokine production, though other T cell functions were not assessed (228). *In vitro*, IDO1 siRNA silencing in a macrophage/CD4^+^ T cell co-culture reduced *Mtb* burden (215). IDO1 silencing in macrophages alone had no effect on *Mtb* burden, indicating IDO1 inhibited anti-mycobacterial T cell functions (215). IDO1 silencing did not increase IFNγ production, though increased IFNβ was detected (215). In *Mtb*-infected macaques, IDO inhibition increased T cell proliferation and memory formation, leading to granuloma reorganization, with CD4^+^ T cell migration to the core (215). Whether CD4^+^ T cell influx into the granuloma is responsible for decreased *Mtb* burden and/or improved *Mtb* clearance has yet to be explored.

The inhibitory effect of IDO on T cells has been attributed to L-TRP depletion. However, metabolites produced by IDO can suppress T_H_1 and T_H_17 responses and promote T_reg_ differentiation, a phenomenon that has been explored in autoimmune diseases (229, 230). As T_reg_ activity inhibits anti-mycobacterial T_H_1 responses, the immunoregulatory role of L-TRP metabolites should be further explored.

#### B Cells

Unlike T cells, L-TRP metabolism in B cells during *Mtb* infection has not been explored, though it has been studied in other infection models. In human PBMCs stimulated with Dengue virus, increasing KYN directly correlated with increased IgG production (231). However, *Ido1* ^-/-^ mice exhibit increased intestinal IgA, which afforded protection against *Citrobacter rodentium* infection (232). KYN and other L-TRP metabolites inhibit splenic B cell antibody production and promote apoptosis (232). In mice, B cell IDO1 is upregulated following T cell-independent antigen immunization, leading to impaired antibody production (233). Further, B cells expressing Epstein-Barr virus latent membrane protein 1 (LMP1) suppressed antibody secretion in LMP1-negative B cells, which was reversed upon 1-MT treatment (234). In a mouse model of *Helicobacter felis*-induced gastritis, *Ido1* ^-/-^ mice displayed decreased B cell frequency within the gastric mucosa (235). Thus, available data on the role of L-TRP metabolism in B cells are conflicting, with the majority of evidence favoring an inhibitory role on the part of IDO.

#### L-Tryptophan Supplementation in Tuberculosis Treatment

Unlike L-ARG, L-TRP supplementation has not been explored in TB. Studies indicate isoniazid (INH) – a front-line *Mtb* antibiotic – interferes with B vitamin metabolism, resulting in low niacin (Vitamin B_3_) levels and development of pellagra (236, 237). In one case study, an HIV^+^ patient showed signs of pellagra after long-term INH prophylaxis, which was reversed upon niacin supplementation and INH discontinuation (237). Considering the link between niacin and L-TRP, it would have been of interest to correlate niacin levels with serum L-TRP, yet this was not measured. Thus, further studies on L-TRP supplementation to prevent pellagra in TB patients taking INH, and subsequent effects on anti-mycobacterial immune responses, are needed.

L-TRP supplementation has been investigated in other infection models. In teleost fish, L-TRP supplementation increased plasma cortisol levels, which decreased circulating leukocytes following *Photobacterium* infection (238). However, infected fish receiving excess dietary L-TRP displayed increased survival (238). In a pig model of rotavirus infection, L-TRP supplementation expanded the Foxp3^+^ T_reg_ population (239). Further studies are needed to determine the effect of L-TRP supplementation on immune responses to *Mtb* infection.

In summary, following infection myeloid cells express IDO, thereby depleting the microenvironment of L-TRP. Similar to L-ARG, T cells require L-TRP to fuel their effector functions, thus setting up yet another competition for nutrients following infection (**Figure 2C**). The role of L-TRP following *Mtb* infection in other immune cells requires further investigation. Additionally, the therapeutic potential of L-TRP supplementation has not yet been explored in the context of *Mtb* infection, but may prove beneficial in patients prescribed INH.

## Concluding Remarks

In this review, we summarized current knowledge on the immune response to *Mtb* and the contribution of immune cell L-ARG and L-TRP metabolism. While availability of other amino acids likely influences immune responses following infection, the L-ARG and L-TRP pathways are the most comprehensive and have identifiable regulatory roles. Available data suggest amino acid metabolism is central in the regulation of immune responses following infection. Infection leads to upregulation of pathways that limit nutrient availability, which in turn inhibits productive immune responses. Effects of nutrient limitation have been most extensively studied in macrophages and T cells, suggesting amino acid restriction by myeloid cells contributes to T cell suppression following *Mtb* infection. However, the immune system must balance amino acid requirements for T cell-mediated *Mtb* control with restriction to suppress T cell-mediated immunopathology. Thus, further research into competition for amino acids between myeloid and T cells should be further explored during *Mtb* infection.

Considering many TB-endemic areas also suffer from malnourishment, continued research into nutrient restriction and its effects on anti-*Mtb* immune responses is needed. Recently, it was shown that latent TB patients with low body mass index have altered immune cell profiles, including decreased memory T and B cells, as compared to latent TB patients with normal body mass index (240). This underscores the importance of nutrient availability in the immune system and parallels studies suggesting nutrient supplementation boosts immune responses following infection. Given their importance in immune cell function, the role of amino acid supplementation should be further explored as a potential therapeutic option in TB, especially in undernourished populations. Better understanding nutrient requirements of immune cells following *Mtb* infection may aid in development of future host-directed therapies aimed to eradicate TB.

## Author Contributions

Both authors contributed to conceptualization and writing (original draft, RC; reviewing and editing, RC and JQ). All authors contributed to the article and approved the submitted version.

## Funding

This work was supported by the National Institutes of Health R01AI116668 (JQ), R21AI148612 (JQ), and the Division of Infectious Diseases at Cincinnati Children’s Hospital Medical Center. The content is solely the responsibility of the authors and does not necessarily represent the views of the funding sources.

## Conflict of Interest

The authors declare the research was conducted in the absence of any commercial or financial relationships that could be construed as a potential conflict of interest.
